# 89 Vasopressor Use and Burn Resuscitation Outcomes at 5 Major US Burn Centers

**DOI:** 10.1093/jbcr/iraf019.089

**Published:** 2025-04-01

**Authors:** Cody McHargue, Tam Pham, Julie Rizzo, James Aden

**Affiliations:** University of Washington; University of Washington; Brooke Army Medical Center; US Army Institute of Surgical Research

## Abstract

**Introduction:**

Prior research indicates that vasopressors are sometimes used during acute burn resuscitation, though significant concerns exist due to vasoconstrictive effects, reduced organ perfusion, and risks of organ failure and death. The purpose of this study was to examine the use of vasopressor infusions (to include epinephrine, norepinephrine, dopamine and vasopressin) during acute burn resuscitation in five major burn centers across the United States.

**Methods:**

This was a secondary analysis of the Burn Navigator (BN) study, a multicenter observational trial that enrolled 300 patients with > 20% TBSA, > 40kg undergoing resuscitation with the BN. Patients who received vasopressors within the first 48 hours were compared to those who did not. To mitigate the differential likelihood of receiving vasopressors, we performed 1:1 propensity-matched analysis for the main outcomes which included fluids received and resuscitation-related complications.

**Results:**

The study cohort included 104 patients (35%) who received vasopressors within 48 hours and 39 (13%) within 6 hours. Vasopressor use varied significantly among the five centers (15-63%, p< 0.001). Vasopressors were used more frequently in patients over 55 years (46% vs. 30%, p= 0.01), and larger TBSA burns (46 ± 19 vs. 35 ± 15, p< 0.001). The propensity-matched analysis revealed that vasopressor use was associated with higher mean primary fluids in the first 24 hours (5.3 vs. 4.4 ml/kg/TBSA, p=0.004), higher AKI incidence (19% vs 7%, P=0.03), and higher 7-day mortality (9% vs. 0%, = 0.007).

**Conclusions:**

Our analysis identified that vasopressor use was associated with worse injury characteristics and worse patient outcomes in critically ill patients undergoing burn resuscitation. This data should prompt the reevaluation of vasopressor use during major burn resuscitation, with the goal to define safe initiation parameters and dosing thresholds, and to minimize resuscitation-related complications.

**Applicability of Research to Practice:**

Very applicable to all critically ill burn patients.

**Funding for the Study:**

Department of Defense, Grant (W81XWH-16-2-0055) awarded to the ABA

